# Pharmacist-Physician Interprofessional Collaboration to Promote Early Detection of Cognitive Impairment: Increasing Diagnosis Rate

**DOI:** 10.3389/fphar.2021.579489

**Published:** 2021-04-27

**Authors:** Hernán Ramos, Juan Pardo, Rafael Sánchez, Esteve Puchades, Jordi Pérez-Tur, Andrés Navarro, Lucrecia Moreno

**Affiliations:** ^1^ Community Pharmacist, Official College of Pharmacists of Valencia, Valencia, Spain; ^2^ Cátedra DeCo MICOF-CEU UCH, Valencia, Spain; ^3^ Embedded Systems and Artificial Intelligence Group, Universidad CEU Cardenal Herrera, Valencia, Spain; ^4^ Department of Neurology, Arnau de Vilanova Hospital, Valencia, Spain; ^5^ Moncada Health Center, Valencia, Spain; ^6^ Unitat de Genètica Molecular, Instituto de Biomedicina de Valencia, CSIC. Centro de Investigación Biomédica en Red en Enfermedades Neurodegenerativas (CIBERNED), Unidad Mixta de Neurología y Genética, Instituto de Investigación Sanitaria La Fe, Valencia, Spain; ^7^ Department of Pharmacy, Universidad CEU Cardenal Herrera, Valencia, Spain

**Keywords:** dementia screening, interprofessional practice, pharmacist-physician, early detection, subjective memory complaints, primary health care

## Abstract

The increased pressure on primary care makes it important for other health care providers, such as community pharmacists, to collaborate with general practitioners in activities related to chronic disease care. Therefore, the objective of the present project was to develop a protocol of action that allows close pharmacist-physician collaboration to carry out a coordinated action for very early detection of cognitive impairment (CI).

**Methods:** A comparative study to promote early detection of CI was conducted in 19 community pharmacies divided into two groups: one group with interprofessional collaboration (IPC) and one group without interprofessional collaboration (NonIPC). IPC was defined as an interactive procedure involving all pharmacists, general practitioners and neurologists. A total of 281 subjects with subjective memory complaints were recruited. Three tests were used in the community pharmacies to detect possible CI: Memory Impairment Screening, Short Portable Mental State Questionnaire, and Semantic Verbal Fluency. Individuals with at least one positive cognitive test compatible with CI, were referred to primary care, and when appropriate, to the neurology service. Finally, we evaluated the differences in clinical and diagnostic follow-up in both groups after six months.

**Results:** The NonIPC study group included 38 subjects compatible with CI referred to primary care (27.54%). Ten were further referred to a neurology department (7.25%) and four of them (2.90%) obtained a confirmed clinical diagnosis of CI. In contrast, in the IPC group, 46 subjects (32.17%) showed results compatible with CI and were referred to primary care. Of these, 21 (14.68%) were subsequently referred to a neurology service, while the remaining 25 were followed up by primary care. Nineteen individuals out of those referred to a neurology service obtained a confirmed clinical diagnosis of CI (13.29%). The percentage of subjects in the NonIPC group referred to neurology and the percentage of subjects diagnosed with CI, was significantly lower in comparison to the IPC group (*p*-value = 0.0233; *p-*value = 0.0007, respectively).

**Conclusions:** The creation of IPC teams involving community pharmacists, general practitioners, and neurologists allow for increased detection of patients with CI or undiagnosed dementia and facilitates their clinical follow-up. This opens the possibility of diagnosis in patients in the very early stages of dementia, which can have positive implications to improve the prognosis and delay the evolution of the disease.

## Introduction

The world’s population is aging fast. The improvements in health care during the last century have contributed to people having longer and healthier lives. However, this extension of life expectancy has also produced an increase in the number of people with age-related diseases, such as dementia, that has resulted in a high demand and pressure on primary care services (World Health Organization [WHO], 2016). This pressure requires the implementation of new strategies to take advantage of all available stakeholders in the healthcare process. For instance, healthcare providers such as community pharmacists can complete many activities associated with care for chronic conditions in the community, relieving the pressure on primary care.

The term “primary care” refers to caring for people rather than simply treating specific diseases or conditions. Primary healthcare can be seen to comprise of three main areas: empowering people and communities, fostering multi-sectoral policy and action, and primary care and essential public health functions as the core of integrated health services (WHO, 2020).

Although Alzheimer’s dementia is clinically diagnosed among adults aged 65 years or more, the pathology begins to develop with brain changes beginning twenty years or earlier before symptoms appear. Researchers have begun to recognize the importance of older adults reporting their own experiences of memory and thinking problems, without (or before) a formal examination by a physician. This personal experience is called subjective cognitive decline and it may indicate an early stage of Alzheimer’s disease (AD) (Alzheimer’s Association, 2019).

With an early detection of cognitive impairment (CI), we could improve the patient's prognosis, ensure the monitoring of their mental health, and slow the evolution of their disease (Morley et al., 2016). In addition, it is well established now that interdisciplinary approaches to health problems result in greater improvement of an individual’s health condition as compared to traditional approaches (Hwang et al., 2017; Saint-Pierre et al., 2018). Therefore, it is pertinent to develop an interdisciplinary preventive action plan to detect these first signs of cognitive change early.

The concept of interprofessional teams has been defined by the WHO in the context of education. However, it is also applicable to clinical practice, and refers to two or more professionals learning or practising together to improve health outcomes (WHO, 2010).

To create an effective action plan in this regard, it is essential to improve interprofessional collaboration (IPC) among primary healthcare stakeholders and study the factors that will ensure the success of such a collaboration (Zwarenstein et al., 2009). An interprofessional practice-based intervention involves the deployment of a tool to foster and improve IPC; examples include communication tools, interprofessional meetings, and checklists (Reeves et al., 2017).

Communication (regular telephone contact and face-to-face communication) is the most cited collaboration factor. Hence, direct, honest, proactive, and regular communication with feedback and information exchange is needed. Trust and respect are other important factors that favor collaboration. This requires an understanding of the role of each profession, mutual recognition, assessment of the other professionals, involvement in the working relationship, and desire to collaborate (Bollen et al., 2019).

The closeness of the pharmacist with his/her neighbors allows one to consider the community pharmacy as a prime instrument to screen the population and identify those who are at early stages of cognitive alteration. We have already shown in an earlier study that this can be achieved by the use of simple neuropsychological tests and lifestyle questionnaires coupled with genetic information (Climent et al., 2018).

In this pilot study, we analyzed, whether the creation of interdisciplinary collaborative teams involving community pharmacists, primary care physicians and neurologists improves the early detection of CI. Our hypothesis is that such a close collaboration would increase both the number of people with CI remitted to a hospital for a diagnosis and the number of subjects diagnosed.

In this paper, we describe the initial results of our project, *Screening for Cognitive Decline* (CRIDECO for its Spanish name). The main objective of CRIDECO is to develop tools that help in the early diagnosis of CI by fostering a close collaboration of the different actors that participate in the public health system, to enable co-ordinated action against CI, thereby giving a better, and earlier response to patients. To the best of our knowledge, this is the first interdisciplinary CI screening in the Valencia Region and we have planned to apply this procedure to the rest of the country.

## Methods

### University Facilitated Interprofessional Interdisciplinary Communication Network: Pharmacist, Primary Care Physician, and Specialist

The multidisciplinary CRIDECO Team of the CEU Cardenal Herrera University has been created to develop a protocol to screen individuals presenting at the community pharmacy with subjective memory complaints, and to subsequently set up a procedure to direct the individuals testing positive, in at least one of the tests, to their general practitioners first and to a neurologist in a hospital for precise diagnosis after that (Climent et al., 2018).

Moreover, the IPC team is provided with educational resources (congress, conferences and meetings) for each of the different phases of the intervention, and project coordinator monthly visits are conducted to get feedback on the team's performance.

Additionally, it needs to be taken care that communication between the different stakeholders is maintained by periodic and diverse communication channels among the director of the health centers, participating pharmacists and chief of neurology department of the hospitals assigned.

**FIGURE 1:**
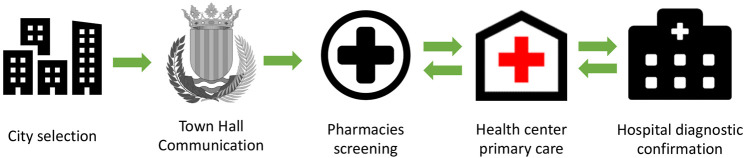
Route to improve interprofessional collaboration into clinical practice to promote IPC group.

On the other hand, a NonIPC group has been created using the same methodology as the IPC group but without carrying out steps 2, 4, and 5 (Table 1), in order to compare results in both groups and assess the importance of creating Pharmacist-Physician interprofessional collaborations to promote early detection of CI (Figure 1).

**TABLE 1:** Five steps of the IPC group protocol.

Step	Target	Action implemented
First: Approaching	Small town	Selection of a small town to be offered the declaration of a neuroprotected city
Second: Engaging	Local government entities and society	Project information to the mayor and dissemination of the project through posters, news in local press, etc.
Third: Training	Community pharmacies	Individual informative visits to join the project
		Training to detect subjective cognitive impairments
		Communicate to the physicians
Fourth: Engaging primary healthcare center units	Primary care	Informative clinical session to all the physicians and pharmacists
		Refer patients to join the study to the pharmacy
		Communicate to the pharmacists
		Diagnosis of patients
		Refer patients to specialist
Fifth: Engaging neurologists	Hospital	Individual informative visit to join the project
		Diagnosis of patients' referrals
		Communicate to investigation team

### Pharmacy and Subject Recruitment

The study was carried out in 19 community pharmacies located in the Valencian region (Spain), over a period of one year (from September 2018 to September 2019). Community pharmacies were divided into two groups, those with an interdisciplinary communication network (IPC; n = 143; 10 pharmacies) and those without it (NonIPC; n = 138; 9 pharmacies). All pharmacies pertained to the Spanish Society of Family and Community Pharmacy and all of them received the same training (1 and 3 steps). However, the geographic distribution in both groups was different which is related to/impacts the reaching of the publicity and dissemination activities. Five subjects were excluded from the study due to their refusal to sign the consent form.

All the community pharmacists (IPC and NonIPC) were first trained by the medical team and the CRIDECO research group prior to performing an active screening to detect cognitive alterations. In both groups during routine dispensing, the pharmacist identified, by express reference of the client or by indirect questions of the pharmacist, signs of subjective memory complaints, appearance of depressive feelings, increased drowsiness, alterations in the recognition of objects, alterations in speech, difficulty in performing certain complex activities such as using public transportation, managing money, and/or following medical treatment. Subjects who presented these signs were invited to join the project.

Individuals meeting the inclusion criteria were informed of the study; signed informed consent was mandatory to participate. The criteria for inclusion were: age of 50 years and above, a subjective complaint of memory, and willingness to participate in the study.

The exclusion criteria were: being under 50 years old, having no subjective complaints, diagnoses of AD or dementia, and severe sensory deficits (blindness, deafness) or physical inability that could have interfered with the ability to complete the tests.

A group of community pharmacies working without the intervention of an IPC team acted as the comparative group (referred to as NonIPC). In both groups, after the screening at the community pharmacy,a report was given to the patient. This report included a brief description of the project and the score obtained in the tests and was handled to the subject instructing them to give it to their primary care physician. The main differences in the IPC group vs. the NonIPC group were (Table 2):

- In the IPC group, the study was publicized in their geographic area. While in the NonIPC group there was no publicity of the study in their location. As both groups were located in different towns, the publicity only reached individuals living in the same area of the community pharmacy in the IPC group.
- In the IPC group, pharmacists and physicians were aware of the project and had defined roles (through joint training and informative clinical sessions). On the other hand, in the NonIPC group only the pharmacists are informed about the existence of the project.
- In the IPC group physicians communicated with pharmacists by letter or face-to-face at informative clinical sessions. However, in the NonIPC group there is no communication between physicians and pharmacists.
- In the IPC group subjects were recruited both by community pharmacy and primary care. That is, in this group physicians referred subjects with subjective complaints to pharmacies in addition of simply receiving individuals with positive results in tests (bidirectionality). On the contrary, in the NonIPC group, subjects were recruited exclusively in the community pharmacy.
- Uniquely in the IPC group physicians wrote code (“CRIDECO”) on the report to neurology that facilitated patient follow-up between departments (neurology-primary care).

**TABLE 2:** Differences in the protocol between NonIPC group and IPC group.

Main differences	NonIPC	IPC
The study was publicized in their geographic area	No	Yes
Physicians were aware of the project and had defined roles	No	Yes
Only the pharmacists are informed about the existence of the project	Yes	No
Physicians communicated with pharmacists by letter or face-to-face at informative clinical sessions	No	Yes
Clinical sessions to share information between pharmacists, primary care physicians and neurologists	No	Yes
Patients were recruited both by community pharmacy and primary care	No	Yes
Subjects were recruited exclusively in the community pharmacy	Yes	No
Physicians wrote code (“CRIDECO”) on the report to neurology that facilitated patient follow-up between departments	No	Yes

**FIGURE 2:**
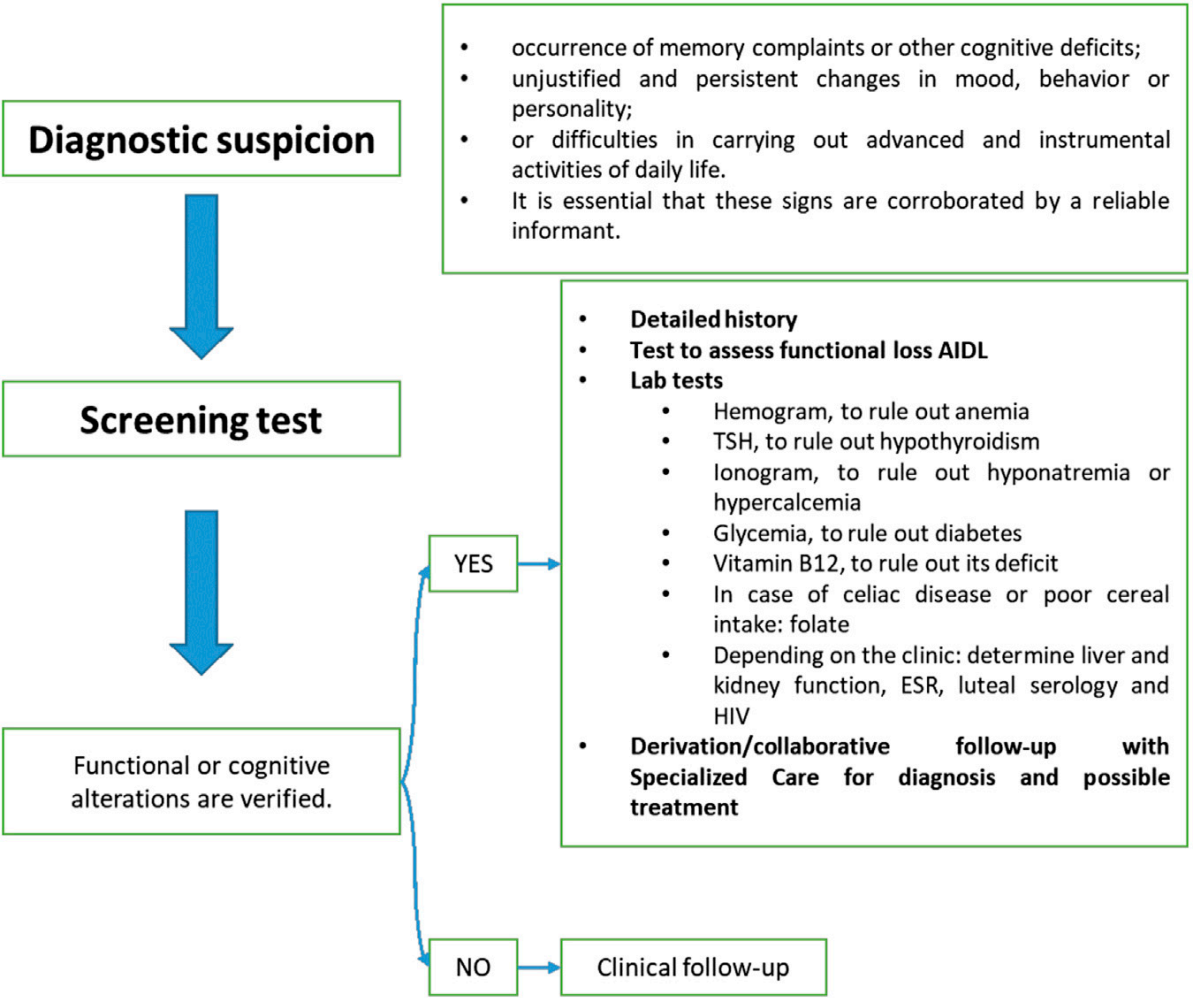
Protocol of action in primary care.

Figure 2 shows the clinical guidelines applied in primary care. Finalizing a definitive diagnosis through specific tests was reserved for specialized care.

### Data Collection and Statistical Treatment

A machine learning technique protocol was used in the community pharmacy to rapidly select candidates for further screening *via* a question-based CI test (Muñoz Almaraz et al., 2020). The idea was to maximize the selection process by attending to those factors that imply a high probability of positive result in the screening tests. For this reason, the subjective memory complaint was used as a criterion for inclusion in the study. On the other hand, the inclusion age (≥50 years) was decided with the aim of detecting patients in the early stages of CI, since most studies of this type are carried out in subjects over 65 years old (Climent et al., 2018).

Then, a questionnaire that included additional study variables such as drug consumption or dietary habits, was completed by the participants in order to collect the largest number of possible factors related to CI for each participant.

Next, the participants were assessed using three validated screening tests: Memory Impairment Screening (MIS) (Böhm et al., 2005), Short Portable Mental State Questionnaire (SPMSQ) (Martínez-De la Iglesia et al., 2001), and Semantic Verbal Fluency (SVF) (López et al., 2013).

#### Memory Impairment Screening

This test assesses verbal learning through reading and subsequent free and facilitated recall of four words, scoring on a 0–8 range. There are several validation studies that have shown acceptable results for cognitive impairment. The MIS uses controlled learning to ensure attention, induce specific semantic processing, and optimize encoding specificity to improve detection of dementia. The MIS also presents a good correlation with the hippocampal and entorhinal volumetric measurements (Buschke et al., 1999; Böhm et al., 2005). It has a sensitivity for dementia of 74% and a specificity of 96%, respectively (Böhm et al., 2005).

#### Short Portable Mental State Questionnaire (Spanish Version)

SPMSQ score is derived from the number of errors based on a 10-item list by coding errors as “1” and correct answers as “0”. Items include tasks on orientation (“What is the date today?”), memory (“What was your mother’s maiden name?”) and attention (“Subtract 3 from 20 and keep subtracting 3 from each new number, all the way down”). Thus, individual cognitive scores ranged from 0 to 10 errors, with lower values indicating better cognitive performance. Already used in previous studies because it is valid for the illiterate population, in addition to its simplicity and wide use in primary care (Schönstein et al., 2019). The sensitivity for detecting CI for this test in Spain was 85.7% and the specificity 79.3% (Martínez de la Iglesia et al., 2001).

#### Semantic Verbal Fluency

Subjects are asked to produce words belonging to a semantic category (e.g., animals) with a limited time (1 min). SFV is widely used in neuropsychological evaluation because is an instrument which is easy and fast to apply. It is very sensitive (74%) and specific (80%), allowing to differentiate with enough precision between subjects with and without dementia at the recommended cut-off score (10 words) (Price et al., 2012; López et al., 2013).

The tests used were chosen after consultation with the Valencian Society of Neurology. The idea behind using these three tests was to detect the maximum possible number of true positives and thus, increase the accuracy of the overall process. Consequently, subjects with a score compatible with the presence of CI in any of the three tests were referred to primary care for medical diagnosis.

We compared for significant differences in efficient detection of participants with a high probability of CI between IPC and NonIPC groups. This was followed by a post-hoc analysis using G*Power statistical software to compute the achieved statistical power of the study, given a significance level of 0.05; the statistical value came out to be 0.96.

After the completion of the follow-up of the subjects, all the information was stored in a database designed specifically for this study. Subsequently, the data was checked by reviewing the subjects’ medical records during the overall process and a posterior data cleansing process was conducted in order to check the completeness and correctness of the dataset. In case there were missing data, not achievable, in order to maximize the value of the sample advanced imputation techniques as MICE (van Buuren et al., 2011) were employed. The statistical analysis was carried out using advanced statistical treatment program R.

### Ethical Approval

The study was reviewed and approved by the Research Ethics Committee of Universidad CEU Cardenal Herrera (approval no. CEI18/027) and by the Research Ethics Committee of Arnau de Vilanova Hospital (MOR-ROY-2018–013). All subjects gave written informed consent in accordance with the Declaration of Helsinki.

## Results

As shown in Table 3, the percentage of women (71.01% in the NonIPC and 72.72% in the IPC group) with respect to men did not differ significantly among the two groups. Similarly, there were no significant differences in the participants’ body mass index (27.80 and 27.97 kg/m^2^ in the NonIPC and IPC groups, respectively), educational level, physical exercise, average weekly reading, and associated comorbidities (diabetes, hypertension, hypercholesterolemia, and depression). However, there were significant differences in the mean age of the NonIPC ($\bar{x}=\;$70.94 years) with respect to the IPC group ($\bar{x}=\;$68.23), probably due to the multiple dissemination activities carried out that encouraged people to enroll in the project and the persistent publicity of the study in the city with IPC group.

**TABLE 3:** Description of quantitative variables in both groups.

Variables		NonIPC (N = 138)	IPC (N = 143)	*p-value*
Subject’s following period (months)		18	18	
Age $\left(\bar{x},\;sd\right)$		70.94 (9.25)	68.23 (8.04)	0.0096
Average BMI (kg/m2) $\left(\bar{x},\;sd\right)$		27.8 (3.72)	27.97 (3.54)	0.4563
Sex [n (%)]	Females	98 (71.01)	104 (72.72)	0.7495
	Males	40 (28.98)	39 (27.27)	
Educational attainment [n (%)]	Illiterate	4 (2.89)	1 (0.69)	0.2031
	Read and write	35 (25.36)	34 (23.77)	
	Primary education	54 (39.13)	71 (49.65)	
	Secondary education	28 (20.28)	27 (18.88)	
	Higher education	17 (12.31)	10 (6.99)	
Weekly physical exercise (h) $\left(\bar{x},\;sd\right)$		4.15 (5.33)	3.28 (3.81)	0.1203
Weekly reading (h) $\left(\bar{x},\;sd\right)$		4.01 (6.00)	2.86 (6.28)	0.1184
Participants with diabetes [n (%)]		38 (27.53)	29 (20.27)	0.1536
Participants with hypertension [n (%)]		78 (56.52)	84 (58.74)	0.7066
Participants with hypercholesterolemia [n (%)]		63 (45.65)	63 (44.05)	0.7880
Participants with depression [n (%)]		38 (27.53)	44 (30.76)	0.5512

IPC, study with interprofessional collaboration; NonIPC, study without interprofessional collaboration.

**FIGURE 3:**
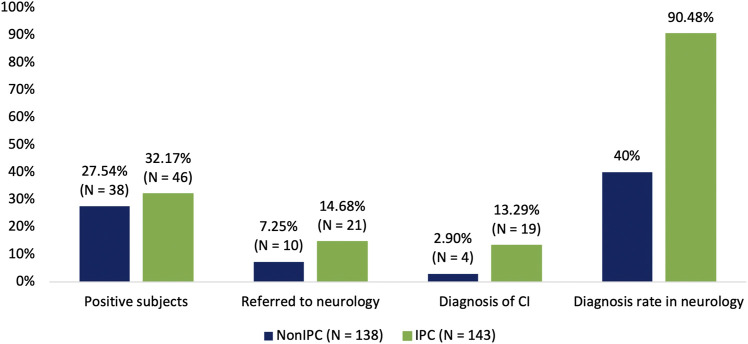
Differences between NonIPC group and IPC group regarding the percentage of positive subjects, the percentage of subjects referred to neurology, and the percentage of subjects diagnosed with CI. The last diagram bar represents the diagnosis rate in neurology in both groups concerning the overall referred subjects.

In order to assess the existing differences in obtaining a diagnosis in neurology in both populations, subjects will be followed up at 24 months, however, the results presented were collected 6 months after the start of the study. At first, the NonIPC group presented 38 positive subjects compatible with CI who were referred to primary care (27.54%). Ten of them were further referred to a neurology department (7.25%) and four of them obtained a confirmed clinical diagnosis of CI (2.90%) (Figure 3). Of the latter, three were diagnosed with mild cognitive impairment (MCI) and one with dementia. The remaining six subjects (60%) were lost to follow-up.

In the IPC group, 46 subjects (32.17%) showed positive results for CI and were referred to primary care. Of these, 21 (14.68%) were subsequently referred to a neurology service, while the remaining 25 were followed up by primary care. Nineteen individuals out of those referred to a neurology service obtained a confirmed clinical diagnosis of CI (13.29%) (Figure 3). Specifically, fourteen individuals were diagnosed as having MCI, 5 individuals were found to have dementia, whereas no information was available for the remaining 2 individuals at the time of this study’s completion.

As it is shown in Table 4 no statistically significant differences were observed in the number of subjects with CI compatible scores in both groups (*p*-value = 0.3965). However, the percentage of subjects referred to neurology is significantly lower in the NonIPC group than in the IPC group (*p*-value = 0.0233). In this context, despite their mean older age, the percentage of diagnosis in neurology over total subjects in both groups was significantly lower in the NonIPC group (*p-*value = 0.0007).

**TABLE 4:** Statistical differences between both groups by using a test of proportions.

Variable	NonIPC (N = 138)	IPC (N = 143)	Test of Proportions *p*-value
Subjects positive in CI tests at community pharmacy [n (%)]	38 (27.54%)	46 (32.17%)	0.3965
Subjects referred to hospital neurology department [n (%)]	10 (7.25%)	21 (14.68%)	0.0233
Subjects diagnosed of CI at hospital neurology department [n (%)]	4 (2.90%)	19 (13.29%)	0.0007

Additionally, the results regarding the number of positive tests in both groups did not report major differences, in the NonIPC group 20 subjects had 1 positive test (14.49%), 13 had two (9.42%) and 5 had all three tests (3.62%). On the other hand, in the IPC group, 22 subjects had 1 positive test (15.38%), 11 had two (7.69%) and 13 had all three tests with positive scores (9.09%). Furthermore, within the group of subjects who tested positive in the neuropsychological tests of the pharmacies, only 10.53% (4 out of 38) of the NonIPC group obtained a diagnosis, while the IPC group was 41.30% (19 out of 46) (Fisher's exact test; *p*-value = 0.0014). This data demonstrates our initial hypothesis that a close collaboration between pharmacists and primary care conducts to a higher number of people with a CI diagnosis.

**FIGURE 4:**
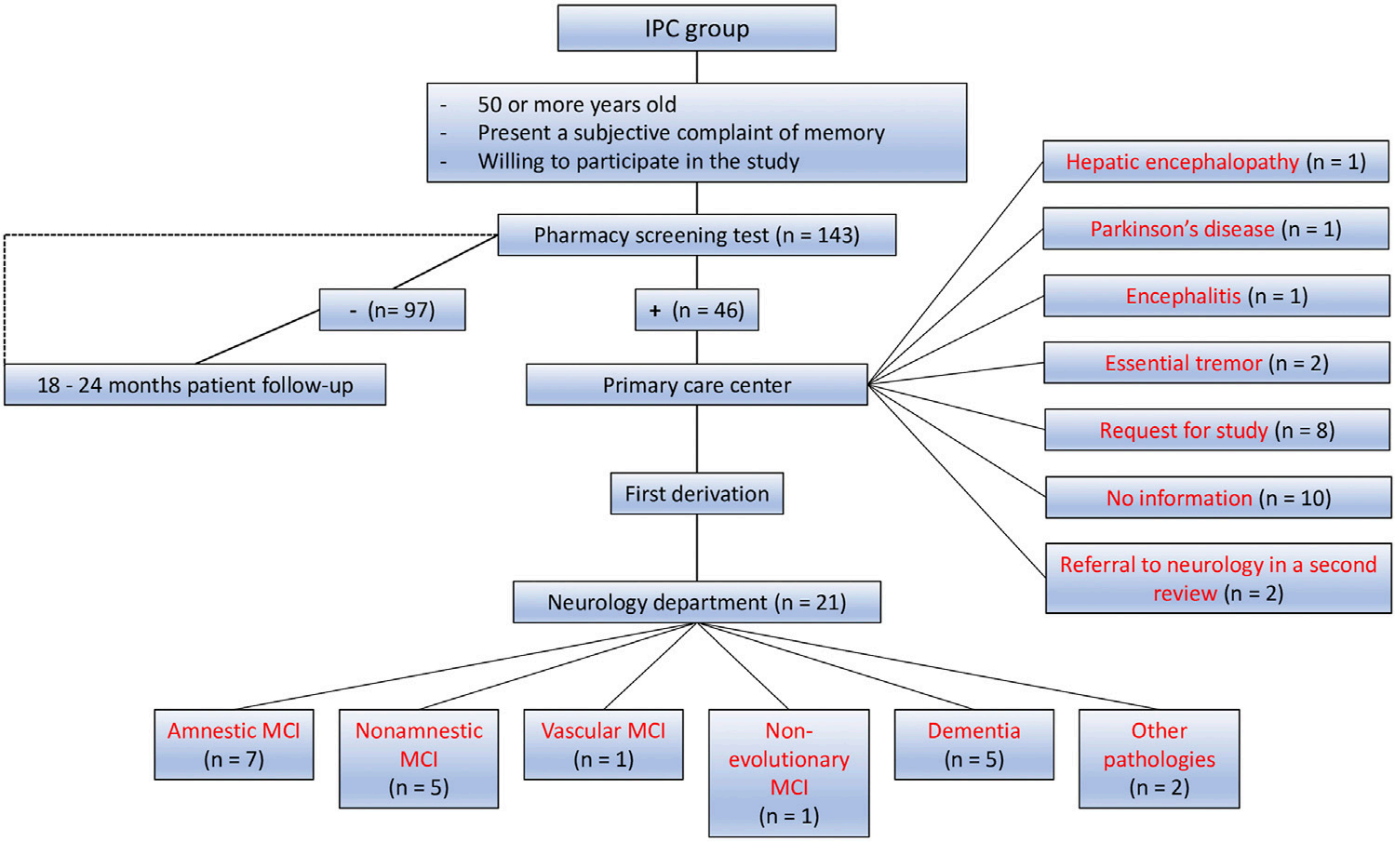
Diagnosis and follow-up of the IPC group at 6 months. The figure shows the diagnoses obtained after 6 months of follow-up in the IPC group, both in primary care and in neurology.

One of the great advances resulting from the work of the interdisciplinary collaborative team was the follow-up of subjects, as can be seen in the flow chart of the IPC city represented in Figure 4. In the IPC group, 46 patients tested positive in pharmacy screening tests out of a total of 143. All of them were referred to primary care. Of those, 21 individuals obtained a diagnosis in the neurology service. The diagnoses obtained in neurology were amnestic MCI (n = 7), non-amnestic MCI (n = 5), vascular MCI (n = 1), non-evolutionary MCI (n = 1), primary degenerative dementia (n = 5), and other pathologies (n = 2). Furthermore, the following diagnoses were obtained from patients examined in primary care: hepatic encephalopathy (n = 1), Parkinson’s disease (n = 1), encephalitis (n = 1), and essential tremor (n = 2). Some patients are currently under study (n = 8), and some others were also referred in a second review, based on results obtained by computed tomography (n = 2). For the remaining subjects (n = 10) their study was not finished in primary care at the time of closing this part of the study (Figure 4).

Another objective was to evaluate the causes of referral to neurology by primary care in the IPC group; the data obtained showed higher mean ages (*p* = 0.0096) and significantly lower mean scores in two screening tests: SVF (*p* = 0.0417), MIS (*p* = 0.0003), to be these causes (Table 5).

**TABLE 5:** Statistical differences regarding referral to neurology in IPC (interprofessional collaboration) group.

Variable		Derivation to neurology		Total	*p-value*
		No	Yes		
N subjects		25	21	46	
Average	Age ($\bar{x}$, sd)	70.08 (5.89)	75.04 (5.60)	72.34	0.0096
	SVF score ($\bar{x}$, sd)	11.36 (4.34)	8.61 (4.47)	10.10	0.0417
	MIS score ($\bar{x}$, sd)	5.52 (2.04)	2.85 (2.48)	4.30	0.0003
	SPMSQ score ($\bar{x}$, sd)	2.76 (1.48)	3.66 (2.46)	3.17	0.1485

Normal range: SVF (≥10), MIS (5–8) and SPMSQ (0–2). CI range: SVF (<10), MIS (<5) and SPMSQ (≥3).

**FIGURE 5:**
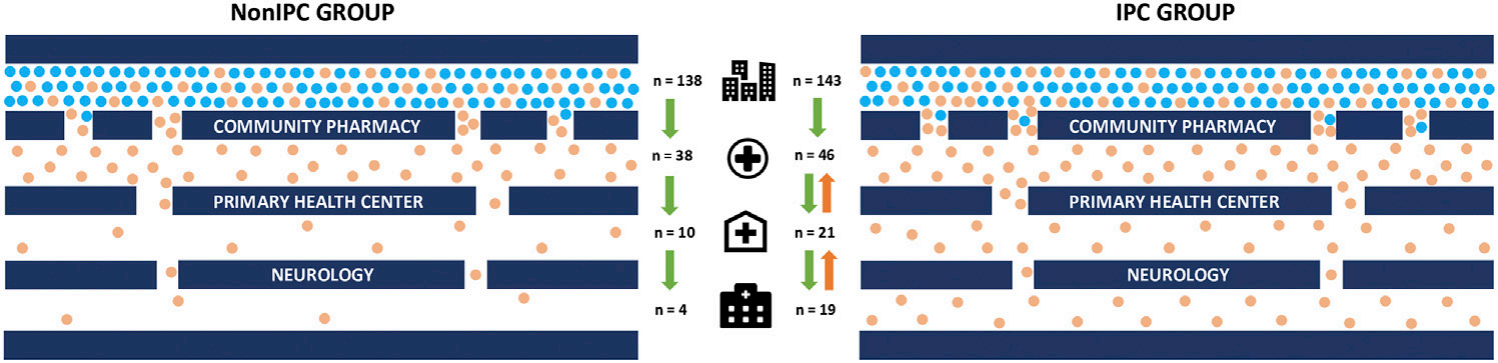
Flowchart of interprofessional collaboration comparing the NonIPC and IPC groups. The green arrows indicate the normal direction of subject referral, while orange arrows represent the bidirectional communication and referral among the different partners of the IPC group. In such group, primary care physicians refer subjects who suspect CI to pharmacies where they are invited to join the project. Whereas neurologists refer individuals without diagnostic to primary care for follow up.

Finally, the two-way interdisciplinary communication between community pharmacists, primary care physicians, and neurology physicians was another great advancement made possible by this study. The presence of this communication network facilitated increased patient flow from the community pharmacy to the specialized neurology area (Figure 5).

## Discussion

There is no cure for Alzheimer's disease, but an early intervention combining pharmacological treatments with cognitive stimulation can delay the progression of the disease and offer a better prognosis and quality of life to patients. However, for the positive effect of both therapeutic approaches to occur, it is essential to perform early interventions, which implies that the condition must be diagnosed in its initial stages (Yu, 2020).

Even though early diagnosis is the only way to implement therapeutical strategies to slow down the progression of AD, it is common for patients to face delays of between 2.8 and 4.7 years to confirm that they have some form of CI (Van Vliet, 2013; Draper, 2016). Mean time from symptom onset to first consultation was in some cases 2.3 years (Draper, 2016). With the current protocol (IPC group) a diagnosis has been achieved in 6 months, with a time from the beginning of the symptoms to first consultation of about 3–6 months. Although it was not initially defined as a specific outcome of our study, it will be of interest to analyze this result once follow-up is completed.

There are cases when patients are diagnosed at such an advanced stage of the disease that the beneficial effect of combining drugs with cognitive stimulation is lost (Prince, 2011).

Every year, in Spain, there are 40,000 new cases of AD, and an additional 12,000–16,000 individuals’ manifest symptoms that can easily be confused as “natural forgetfulness”. Although the disease has no cure, there are treatments that, at least for a limited period of time, manage to stop or slow down the progression of the disease, especially if applied early in the degenerative process. Therefore, early treatment can stabilize the affected person in the milder phases of the disease and delay the evolution of the disease by a few months or years, which is essential to improve the quality of life of the patients (Prince, 2011). Considering that at a given time, 80% of all AD cases are still in the mild phase (WHO, 2017), ensuring successful intervention is important.

Several studies have shown that people with subjective cognitive decline have an increased risk of progressing to dementia. Furthermore, there is evidence that this group has a higher prevalence of positive biomarkers for amyloidosis and neurodegeneration. Consequently, these findings support the idea that subjective cognitive complaints can be an early clinical marker of pre-dementia stages (Studart, 2016). Prevalence rates of CI range from 4 to 9% in most studies including subjects older than 65 years (Villarejo, 2019). In our population, applying a decision tree model (Climent et al., 2018) resulted in the identification of a significant number of individuals presenting with subjective memory complaints, increasing the detection percentage of CI to 30% and maximizing the rate of possible CI cases; because of this, we were able to achieve an early diagnosis.

In order to mitigate the economic barriers to obtaining preventive services, there is a growing interest in taking advantage of interprofessional teams that are positioned to address practices related to prevention, population health management, care coordination, and access to medical care (Fowler 2020).

One powerful barrier to early detection in primary care is the lack of collaborative practice, and an important collaboration to consider is that with the community pharmacist. There is evidence pointing to the significant positive impact of including pharmacists as members of interdisciplinary teams in the assessment of the appropriateness of medications, medication adherence, vascular risk factors; or in controlling diabetes, hypertension or hyperlipidemia (Milosavljevic, 2018). This collaboration also helps to identify drug-related problems resulting in cost savings for the polypharmacy elderly. Moreover, a recent cross-sectional study using the AD knowledge survey among Spanish pharmacists and physicians reported that knowledge in both professional collectives is high, with scores of above 80% in the categories of diagnosis, treatment, and symptoms (Alacreu et al., 2019). These data reinforce the pharmacist’s potential to collaborate in CI detection.

Furthermore, in the NonIPC group, due to higher mean age, a higher detection of CI would be expected. However, interdisciplinary collaboration (IPC group) has boosted a higher patient detection with respect to the NonIPC group, which reinforces our hypothesis. We postulate that the lower mean age in the IPC group may be due to higher participation in the study of younger patients, probably due to greater publicity and diffusion of the study.

This is the first study describing the inclusion of the community pharmacist in the interdisciplinary healthcare team detecting CI. The results of this collaborative intervention are very striking in the IPC group, as almost half of the patients who arrive at the health center are referred to neurology and we know the clinical diagnosis and the reasons for non-remission of the remaining patients at this moment. In the NonIPC group, only a quarter of the patients arriving at the health center are referred and we are not aware of the reasons regarding why the remaining patients are not referred. Interprofessional communication facilitates the outcome of 4 times more diagnosed cases in the hospital as compared to screening carried out without physician-pharmacist collaboration. Moreover, we have a neurology diagnosis rate of 90% as compared to 40% when there is no collaboration. For this reason, we consider the community pharmacy as the entry point in the healthcare system and propose taking advantage of its closeness to the individual as well as the close pharmacist-patient relationship. The results of this study are corroborated by a systematic review and meta-analysis published in 2010 with 298 studies, which concluded that direct patient care provided by the pharmacist has favourable effects on patient outcomes and that incorporating pharmacists as members of the healthcare team is one solution to improve medical care (Chisholm-Burns, 2010).

### Limitations of the Study

The number of participants and the area of application are our limitations. This is a pilot study designed to be a *proof of concept* in order to obtain enough information about the feasibility of the process we designed. In this respect, we plan to extend the protocol to a wider geographical area. So that, in the long term it could be implemented in other Spanish cities.

## Conclusion

In recent years, medical care for dementia has based its efforts on early detection, due to the many benefits reported when there is a clinical diagnosis in the early stages of Alzheimer’s disease. For this reason, the inclusion of community pharmacists in the early detection system for this pathology is a very important tool for achieving this end, mainly because of the accessibility of this group. This study demonstrates the effectiveness of collaboration when both health professionals work with a common objective. The data obtained show that the sensitivity in the screening of CI of those patients who came to the neurology service was very high (90%), thus endorsing the capacity to perform a very sensitive screening in the community pharmacy. On the other hand, the inclusion of this group means an additional help for those patients with subjective memory complaints not detected by the national health system. The creation of interprofessional collaboration teams allows the optimization of monitoring, helps the detection of undiagnosed patients, and increases the clinical information of the patient in both collectives. This allows us to provide a more individualized and comprehensive treatment to the patient, the focus of our attention.

## Data Availability

The raw data supporting the conclusions of this article will be made available by the authors, without undue reservation.

## References

[B1] Alacreu M. Pardo J. Azorín M. Climent M. T. Gasull V. Moreno L. (2019). Importance of Increasing Modifiable Risk Factors Knowledge on Alzheimer's Disease Among Community Pharmacists and General Practitioners in Spain. Front. Pharmacol. 10, 860. 10.3389/fphar.2019.00860 31474852 PMC6704342

[B2] Alzheimer’s Association (2019). Alzheimer’s Disease Facts and Figures. Alzheimers Dement. 15 (3), 321–87. 10.1016/j.jalz.2019.01.010

[B4] Böhm P. Peña-Casanova J. Gramunt N. Manero R. M. Terrón C. Quiñones-Ubeda S. (2005). Spanish Version of the Memory Impairment Screen (MIS): Normative Data and Discriminant Validity. Neurologia. 20, 402–411. 16217689

[B5] Bollen A. Harrison R. Aslani P. van Haastregt J. C. M. (2019). Factors Influencing Interprofessional Collaboration between Community Pharmacists and General Practitioners-A Systematic Review. Health Soc. Care Community 27, e189–e212. 10.1111/hsc.12705 30569475

[B6] Buschke H. Kuslansky G. Katz M. Stewart W. F. Sliwinski M. J. Eckholdt H. M. (1999). Screening for Dementia with the Memory Impairment Screen. Neurology 52, 231–238. 10.1212/wnl.52.2.231 9932936

[B7] Buuren S. V. Groothuis-Oudshoorn K. (2011). Mice: Multivariate Imputation by Chained Equations inR. J. Stat. Soft. 45, 1–67. 10.18637/jss.v045.i03

[B8] Chisholm-Burns M. A. Kim Lee J. Spivey C. A. Slack M. Herrier R. N. Hall-Lipsy E. (2010). US Pharmacists' Effect as Team Members on Patient Care. Med. Care. 48, 923–933. 10.1097/MLR.0b013e3181e57962 20720510

[B9] Climent M. T. Pardo J. Muñoz-Almaraz F. J. Guerrero M. D. Moreno L. (2018). Decision Tree for Early Detection of Cognitive Impairment by Community Pharmacists. Front. Pharmacol. 9, 1232. 10.3389/fphar.2018.01232 30420808 PMC6215965

[B10] Draper B. Cations M. White F. Trollor J. Loy C. Brodaty H. (2016). Time to Diagnosis in Young-Onset Dementia and its Determinants: the INSPIRED Study. Int. J. Geriatr. Psychiatry. 31 (11), 1217–1224. 10.1002/gps.4430 26807846

[B11] Fowler T. Garr D. Mager N. D. P. Stanley J. (2020). Enhancing Primary Care and Preventive Services through Interprofessional Practice and Education. Isr. J. Health Pol. Res. 9, 12. 10.1186/s13584-020-00371-8 PMC7092466 32204734

[B12] Hwang A. Y. Gums T. H. Gums J. G. (2017). The Benefits of Physician-Pharmacist Collaboration. J. Fam. Pract. 66, E1–E8. 29202145

[B13] López A. G. Calero M. D. Navarro-González E. (2013). Predicción del deterioro cognitivo en ancianos mediante el análisis del rendimiento en fluidez verbal y en atención sostenida. Rev. Neurol. 56, 1–7. 10.33588/rn.5601.2012281 23250675

[B14] Martínez de la Iglesia J. Dueñas R. Onís M. C. Aguado C. Albert C. Luque R. (2001). Adaptación y validación al castellano del cuestionario de Pfeiffer (SPMSQ) para detectar la existencia de deterioro cognitivo en personas mayores de 65 años. Med. Clin. (Barc). 117, 129–134. 10.1016/s0025-7753(01)72040-4 11472684

[B15] Milosavljevic A. Aspden T. Harrison J. (2018). Community Pharmacist-Led Interventions and Their Impact on Patients' Medication Adherence and Other Health Outcomes: a Systematic Review. Int. J. Pharm. Pract. 26, 387–397. 10.1111/ijpp.12462 29927005

[B17] Morley J. E. Morris J. C. Berg-Weger M. Borson S. Carpenter B. D. del Campo N. (2015). Brain Health: The Importance of Recognizing Cognitive Impairment: An IAGG Consensus Conference. J. Am. Med. Directors Assoc. 16, 731–739. 10.1016/j.jamda.2015.06.017 PMC4822500 26315321

[B18] Muñoz-Almaraz F. J. Climent M. T. Guerrero M. D. Moreno L. Pardo J. (2020). A Machine Learning Approach to Design an Efficient Selective Screening of Mild Cognitive Impairment. J Vis Exp. 155, e59649. 10.3791/59649 31984961

[B19] Price S. E. Kinsella G. J. Ong B. Storey E. Mullaly E. Phillips M. (2012). Semantic Verbal Fluency Strategies in Amnestic Mild Cognitive Impairment. Neuropsychology 26, 490–497. 10.1037/a0028567 22746308

[B20] Prince M. Bryce R. Ferri C. (2011). World Alzheimer Report 2011. The Benefits of Early Diagnosis and Intervention, London: Alzheimer Disease International. https://www.alz.co.uk/research/WorldAlzheimerReport2011.pdf

[B21] Reeves S. Pelone F. Harrison R. Goldman J. Zwarenstein M. (2017). Interprofessional Collaboration to Improve Professional Practice and Healthcare Outcomes. Cochrane Database Syst. Rev. 6, CD000072. 10.1002/14651858.CD000072.pub3 28639262 PMC6481564

[B22] Saint-Pierre C. Herskovic V. Sepúlveda M. (2018). Multidisciplinary Collaboration in Primary Care: A Systematic Review. Fam. Pract. 35, 132–141. 10.1093/fampra/cmx085 28973173

[B23] Schönstein A. Wahl H.-W. Katus H. A. Bahrmann A. (2019). SPMSQ for Risk Stratification of Older Patients in the Emergency Department. Z. Gerontol. Geriat. 52, 222–228. 10.1007/s00391-019-01626-z PMC6821671 31620876

[B24] Studart A. Nitrini R. (2016). Subjective Cognitive Decline: The First Clinical Manifestation of Alzheimer's Disease?. Dement. Neuropsychol. 10 (3), 170–177. 10.1590/S1980-5764-2016DN1003002 29213452 PMC5642412

[B25] Van Vliet D. De Vugt M. E. Bakker C. Pijnenburg Y. A. L. Vernooij-Dassen M. J. F. J. Koopmans R. T. C. M. (2013). Time to Diagnosis in Young-Onset Dementia as Compared with Late-Onset Dementia. Psychol. Med. 43, 423–432. 10.1017/S0033291712001122 22640548

[B26] Villarejo A. Eimil M. Llamas S. Llanero M. López de Silanes C. Prieto C. (2017). Report by the Spanish Foundation of the Brain on the Social Impact of Alzheimer Disease and Other Types of Dementia. Neurologia. 36, 39–49. 10.1016/j.nrl.2017.10.005 29249303

[B27] World Health Organization (2010). Framework for Action on Interprofessional Education and Collaborative Practice, Geneva: WHO. 2010. 21174039

[B28] World Health Organization (2016). World Health Statistics 2016: Monitoring Health for the SDGs Sustainable Development Goals. Geneve, Switzerland: World Health Organization Retrieved from https://www.who.int/gho/publications/world_health_statistics/2016/en/.

[B29] World Health Organization (2017). Global Action Plan on the Public Health Response to Dementia 2017-2025. Geneve, Switzerland: World Health Organization (2020). Primary health care Available at https://www.who.int/news-room/fact-sheets/detail/primary-health-care (Accessed May 5, 2020)

[B30] Yu J.-T. Xu W. Tan C.-C. Andrieu S. Suckling J. Evangelou E. (2020). Evidence-based Prevention of Alzheimer's Disease: Systematic Review and Meta-Analysis of 243 Observational Prospective Studies and 153 Randomised Controlled Trials. J. Neurol. Neurosurg. Psychiatry. 91, 1201–1209. 10.1136/jnnp-2019-321913 32690803 PMC7569385

[B31] Zwarenstein M. Goldman J. Reeves S. (2009). Interprofessional Collaboration: Effects of Practice-Based Interventions on Professional Practice and Healthcare Outcomes. Cochrane Database Syst. Rev. 3, CD000072. 10.1002/14651858.CD000072.pub2 19588316

